# Bioinspired Modifications of PEEK Implants for Bone Tissue Engineering

**DOI:** 10.3389/fbioe.2020.631616

**Published:** 2021-01-12

**Authors:** Xinming Gu, Xiaolin Sun, Yue Sun, Jia Wang, Yiping Liu, Kaixuan Yu, Yao Wang, Yanmin Zhou

**Affiliations:** Department of Oral Implantology, Hospital of Stomatology, Jilin University, Changchun, China

**Keywords:** polyetheretherketone, surface modification, implants, bioactivity, osseointegration

## Abstract

In recent years, polyetheretherketone (PEEK) has been increasingly employed as an implant material in clinical applications. Although PEEK is biocompatible, chemically stable, and radiolucent and has an elastic modulus similar to that of natural bone, it suffers from poor integration with surrounding bone tissue after implantation. To improve the bioactivity of PEEK, numerous strategies for functionalizing the PEEK surface and changing the PEEK structure have been proposed. Inspired by the components, structure, and function of bone tissue, this review discusses strategies to enhance the biocompatibility of PEEK implants and provides direction for fabricating multifunctional implants in the future.

## Introduction

Bone defects caused by aging, trauma, disease, congenital abnormalities, and surgical resections are widespread all over the world. It's reported that the number of fractures is estimated to rise from 2.1 million to over 3 million during 2005–2025 in the United States (Quarto and Giannoni, 2016). In the past decades, numerous biomaterials have been employed to repair bone defects. Conventional implants used in bone tissue engineering are commonly made of titanium (Ti) or its alloys due to their good biocompatibility, chemical stability, and mechanical properties. However, these materials suffer from limitations such as the release of harmful metal ions, osteolysis, allergenic effects, and radiopaqueness (Niki et al., 2001; Fage et al., 2016). In particular, Ti possesses an elastic modulus of more than 100 GPa, which contributes to stress shielding and the resorption of surrounding bone (Huiskes et al., 1992). To overcome these drawbacks and reduce negative post-implantation biological reactions, polyetheretherketone (PEEK) and PEEK-based compounds have emerged as viable alternatives to Ti and its alloys.

PEEK, which is a dominant member of the polyaryletherketone (PAEK) family, was first synthesized by British scientists via nucleophilic displacement in 1972 (Eschbach, 2000). In the late 1990s, PEEK was approved by the U.S. Food and Drug Administration as an implantable biomaterial (Kurtz and Devine, 2007). Since then, PEEK has been extensively employed in the fields of orthopedics, trauma, spinal and dental implants. Another important member of PAEK named polyetherketoneketone (PEKK), has also been extensively studied as a promising orthopedic implant recently (Wang et al., 2017; Yuan et al., 2018). PAEK has numerous beneficial characteristics such as non-toxicity, excellent mechanical properties, natural radiolucency, and good chemical and sterilization resistance (Wenz et al., 1990; Katzer et al., 2002; Godara et al., 2007). However, smooth PEEK implants can lead to poor osseointegration characterized by fibrous encapsulation, potentially leading to clinical failure (Walsh et al., 2015). This phenomenon can be explained by the hydrophobic and chemically inert properties of PEEK. Consequently, considerable efforts should focus on modifying the surfaces of PEEK implants. It is expected that the reinforced PEEK implants can result in enhanced ingrowth of osteoblasts (osteoconduction), direct contact with surrounding bone (osseointegration) and stimulation of immature cells into osteogenic cells (osteoinduction).

**Figure 1:**
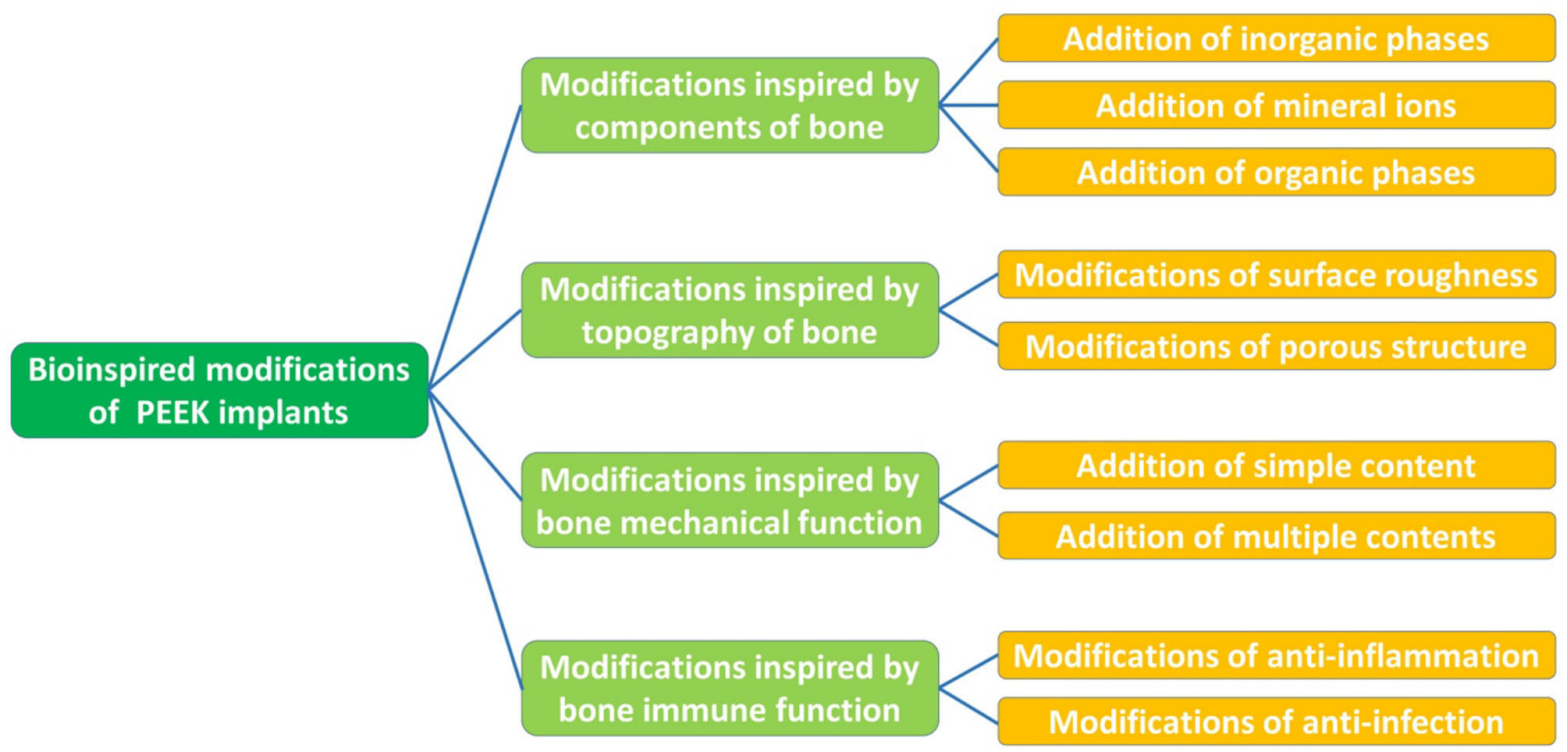
Summary of bioinspired modifications of PEEK implants.

This review presents an overview of the properties of PEEK and multiple strategies for the modification of PEEK implants inspired by the constituents, structure, and function of human bone (Figure 1). Subsequently, we discuss several aspects in need of further exploration and highlight future directions in the field of multifunctional implants.

## Properties of Peek

### Bulk Properties

Bulk properties are determined by the atomic composition and structure of a material (Binyamin et al., 2006), including the mechanical, chemical, thermal, and radiation characteristics, which are fundamental to the performance of an implant. PEEK is a semi-crystalline linear polycyclic aromatic thermoplastic that has an aromatic molecular backbone interconnected by ketone and ether functional groups between the aryl rings (Kurtz and Devine, 2007). Regarding its mechanical behavior, the tensile modulus, bending modulus, and compressive modulus of PEEK are ~3.8, 3.6, and 2.8 GPa, respectively (Han et al., 2019b). At room temperature, PEEK is chemically stable in all conventional solvents except for 98% sulfuric acid (Ha et al., 1997). At high temperatures, PEEK maintains stability and displays resistance to damage from chemical components and radiation. The glass transition temperature of PEEK is ~143°C, whereas the crystalline melt transition occurs at around 343°C (Kurtz and Devine, 2007). In addition, gamma irradiation can be used to sterilize PEEK in air at doses of 25–40 kGy in clinical practice (Kurtz and Devine, 2007).

### Surface Properties

The interactions that occur at the interface of a material define its surface properties (Binyamin et al., 2006). For PEEK implants, one of the key surface properties is biocompatibility. A previous study showed that PEEK has no cytotoxic or mutagenic effects (Katzer et al., 2002). Moreover, human gingival fibroblasts (HGFs) and osteoblasts adhered on the surface of PEEK displayed increased proliferation and viability compared to those on Ti, indicating that PEEK exhibits desirable performance with both soft and hard tissues (da Cruz et al., 2019). Other surface properties, including surface topography, chemistry, energy, and wettability, should also be considered. Surface topography and chemistry can influence the tissue response to an implant by altering protein adsorption and subsequent cell adhesion and differentiation. The surface energy has been confirmed to affect cell maturation, differentiation, and osseointegration (Kilpadi and Lemons, 1994; Zhao et al., 2005). Surfaces with higher surface energy exhibit more rapid cell activation and differentiation than those with lower surface energy. Unmodified PEEK with a water contact angle of 80–90° is inherently hydrophobic and bioinert. When the surface of an implant becomes hydrophilic, plasma proteins and cells are more likely to adhere to it (Jimbo et al., 2010). Surface wettability is related to surface roughness, Elawadly et al. proposed that the water contact angle of PEEK-based materials was below 90° when the surface average roughness (Ra) value was either <1.0 or> 1.7 μm (Elawadly et al., 2017).

## Modifications Inspired by The Components of Bone

### Addition of Inorganic Phases

Human bone is mainly composed of inorganic and organic phases. Calcium phosphate (CaP), which is the primary inorganic constituent of human bone, has been extensively studied in bone tissue engineering owing to its superb biocompatibility, osseointegration, osteoconduction, and osteoinductivity (Eliaz and Metoki, 2017). CaP can enhance the local concentrations of calcium and phosphate ions and form apatite on the surface of the substrate. Meanwhile, CaP can adsorb extracellular matrix (ECM) proteins and activate osteoblastic differentiation via cell–ECM interactions (Eliaz and Metoki, 2017). CaP coatings have been shown to remarkably enhance the proliferation of MC3T3-E1 cells and apatite-forming ability in simulated body fluid (SBF) (Jabbari et al., 2018). In another study, surface functionalization with a combination of phosphate and calcium remarkably promoted the osteogenic activity of rabbit bone marrow-derived mesenchymal stem cells *in vitro* and the osseointegration ability *in vivo* (Sunarso et al., 2019). It is worth noting that amorphous calcium phosphate can be fabricated on the PEEK surface within 1 day, and strong adhesion with PEEK can be obtained using a novel PrA treatment (involving three steps: H_2_SO_4_ treatment, O_2_ plasma treatment, and alkaline SBF treatment) (Yabutsuka et al., 2017, 2018). Further study revealed that the PrA treatment resulted in zero cytotoxicity and produced excellent bone-bonding properties (Masamoto et al., 2019). However, the use of CaP-based materials in high-load-bearing areas is limited due to the poor mechanical properties of CaP (Ambard and Mueninghoff, 2006).

**Figure 2:**
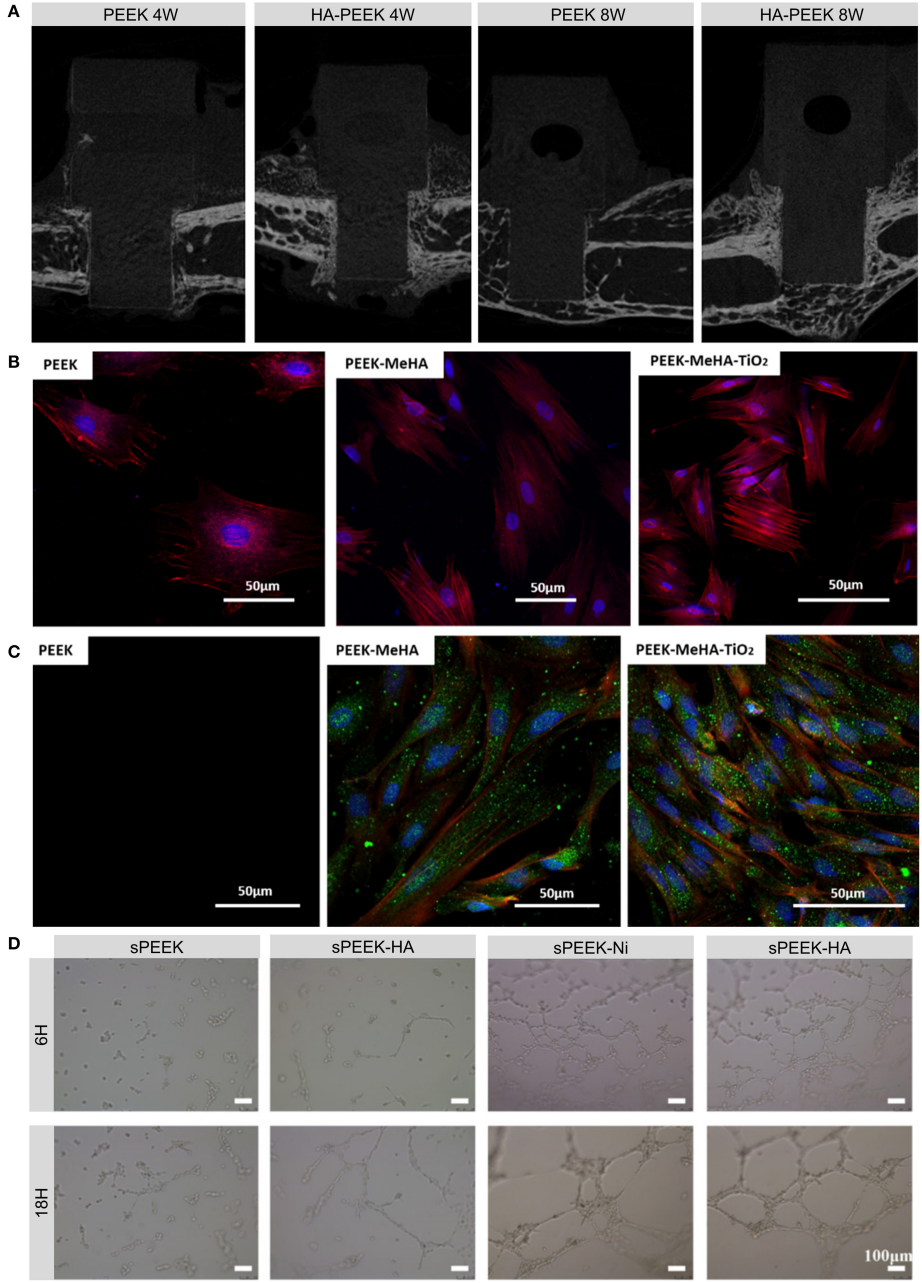
Examples and biological effects of modifications inspired by the components of bone. **(A)** Micro-CT images of new bone formed on HA-coated PEEK and on uncoated PEEK at 4 and 8 weeks after implantation. **(B)** Fluorescence micrographs of actin (red) and nucleus (blue) in rBMSCs grown on PEEK, PEEK-MeHA, and PEEK-MeHA-TiO_2_ for 24 h. **(C)** Immunofluorescence analysis of actin (red), collagen I (green), and nucleus (blue) in rBMSCs grown on PEEK-MeHA and PEEK-MeHA-TiO_2_ for 7 days. **(D)** HUVEC tube formation ability in extracts of sulfonated PEEK (sPEEK), sPEEK-HA, sPEEK-Ni, and sPEEK-Ni-HA at 6 and 18 h. Reproduced with permission from Dong et al. (2020). Copyright (2020) American Chemical Society.

Hydroxyapatite (HA) is widely used in bone regeneration since its composition is close to that of natural bone (Yoshikawa and Myoui, 2005). HA is the most stable and least soluble CaP ceramic and has the molecular formula Ca_10_(PO_4_)^6^ (OH)_2_, in which the Ca/P ratio is approximately 1.67 (Klein et al., 1990; He et al., 2003). HA is considered to be osteoconductive but not osteoinductive (Samavedi et al., 2013). HA can be used as either a coating or a reinforcement component to produce bioactive PEEK composites. Coating PEEK with HA can impart PEEK with a rough and hydrophilic surface, which is beneficial for cell growth (Figure 2A; Lee et al., 2013). Numerous treatments including cold spraying, plasma spraying, ion beam-assisted deposition, and electrophoretic co-deposition have been adopted to deposit HA coatings (Lee et al., 2013; Suska et al., 2014; Durham et al., 2016; Baştan et al., 2018). Unfortunately, plasma-sprayed HA coatings suffer from insufficient adhesion strength to PEEK and tend to delaminate, resulting in the infiltration of multinucleated giant cells (Reigstad et al., 2011). In addition, the detachment of thick, layered apatite coatings was found to cause severe inflammatory response and bone resorption (Røkkum et al., 2003). To suppress such negative responses, a thin nanosized HA (nHA) layer was applied to the PEEK surface. *In vivo* studies indicated that this nHA coating significantly improved the removal torque, early bone integration, and osteoconductive properties of PEEK implants (Barkarmo et al., 2013; Johansson et al., 2014, 2015, 2016, 2018). Chemical modifications such as the grafting of phosphonate groups have also performed positive effects on the adhesion strength between HA and PEEK implants (Mahjoubi et al., 2017).

Subsequent studies attempted to develop bioactive PEEK composites by adding HA, nHA, or ion-substituted HA to PEEK (Wong et al., 2009; Deng et al., 2015b; Liu et al., 2016; Ma and Guo, 2019; Bastan, 2020; Dong et al., 2020). For instance, the addition of strontium-containing HA not only improved the bending modulus of PEEK, it also enhanced apatite formation in SBF and cell-mediated mineralization *in vitro* (Wong et al., 2009). Moreover, increasing the HA content can improve the elastic modulus, compressive strength, and hardness of the composite but not the tensile strength. A HA/PEEK composite containing 30 wt% HA exhibited a higher elastic modulus and slightly lower tensile strength than that of pure PEEK (Ma and Guo, 2019). To optimize the mechanical properties of HA/PEEK hybrid materials, researchers have focused on incorporating reinforcement components [e.g., carbon fiber (CF) and carbon nanotubes (CNTs)] and identifying the optimal content of HA (Deng et al., 2015b; Liu et al., 2016).

### Addition of Mineral Ions

To evaluate the biological properties of PEEK implants, it is crucial to choose biomaterials with good osteogenesis ability for bone repair. Metal ions are strong candidates as coating materials for PEEK implants. Han et al. proposed that electron beam deposition could be used to deposit a Ti layer on the surface of PEEK with strong adhesion and enhanced wettability. Compared to PEEK alone, the Ti-coated PEEK implants exhibited two times greater proliferation and differentiation of MC3T3-E1 cells along with better osseointegration ability (Han et al., 2010). Ti plasma spraying, another coating process, increased the microscale surface area by 40% and accelerated cement line formation in human osteoprogenitor cells (hFOB 1.19) while also increasing the shear strength and bone integration at the implant surface (Walsh et al., 2015; Hickey et al., 2019). Moreover, the addition of Ti increased the compressive strength and stiffness of PEEK (Jung et al., 2016).

Titanium dioxide (TiO_2_) can also be deposited onto the surface of PEEK using several coating methods. Before sol-gel coating, pretreatment with UV, O_2_ plasma, or sandblasting can enhance the bonding strength of the coating layer, while post-treatment with HCl can induce apatite formation, which is related to the bone-bonding ability *in vivo* (Kizuki et al., 2015; Shimizu et al., 2016). Different TiO_2_ nanostructures such as nanoparticles, nanotubes, and nanofibers can also be employed to improve the cytocompatibility, soft tissue integration, and osseointegration of PEEK implants due to their favorable bioactivity and surface morphology (Figures 2B,C; Wu et al., 2012; Lu et al., 2014; Wang X. et al., 2016; Liu et al., 2018).

Other coating materials such as tantalum, tantalum pentoxide, and niobium pentoxide can not only elevate the surface energy, surface roughness, hydrophilicity, protein absorption, and mechanical properties of PEEK implants, they can also induce positive cellular responses and osteointegration (Lu et al., 2015; Mei et al., 2019; Ge et al., 2020). Silicon-based compounds have been found to reinforce the mechanical properties, surface hydrophilicity, apatite mineralization, and cell and bone tissue responses (Ma et al., 2014; Wen et al., 2016; Monich et al., 2017; Zhang et al., 2018a). Other additives such as phosphonate, amorphous magnesium phosphate, graphene, and diamond-like carbon were found to exert similar effects (Wang et al., 2010; Mahjoubi et al., 2017; Ren et al., 2018; Yan J. H. et al., 2018; Sikder et al., 2020).

### Addition of Organic Phases

The organic phase of bone tissue includes a plethora of growth factors and proteins that play a prominent role in osteogenesis and angiogenesis. Bone morphogenetic protein-2 (BMP-2), which is one of the strongest osteoinductive factors, can initiate the differentiation of mesenchymal stem cells (MSCs) into osteoblasts and promote osteogenesis (Ryoo et al., 2006). Therefore, to improve the biological behavior of PEEK, researchers have attempted to immobilize BMP-2 on PEEK through several methods, including the deposition of nanoporous TiO_2_ layers, polyelectrolyte multilayer films, and phosphorylated gelatin coatings along with sulfonation treatment (Han et al., 2014; Guillot et al., 2016; Sun et al., 2018; Wu et al., 2018). It was reported that BMP-2-coated PEEK with a dose of 9.3 μg could lead to localized and temporary bone impairment (Guillot et al., 2016). Thereby, further studies should focus on determining the optimal dose of BMP-2 to maximize osteogenic activity. Functionalizing cell-interfacing surfaces with cell-modulatory proteins can also improve the biological properties of the implant. For example, the ECM protein tropoelastin has been shown to promote the biological behavior of human osteoblast-like osteosarcoma cells (SAOS-2) on PEEK via plasma immersion ion implantation (PIII) treatment (Wakelin et al., 2018). Adiponectin (APN), which is adipocyte-secreted adipokine, has been confirmed to increase the osteogenic ability *in vitro* and osseointegration *in vivo* of sPEEK implants (Wang et al., 2019; Deng et al., 2020). Out of these proteins, bone forming peptide (BFP) can also be utilized to improve the osteogenic differentiation and maturation of MG-63 cells (Wang et al., 2018).

In addition to the aforementioned osteoinductive agents, angiogenic growth factors such as vascular endothelial growth factor (VEGF), transforming growth factor-β, placental growth factor, and fibroblast growth factor can regulate angiogenesis, which involves endothelial cell proliferation, migration, and tube formation (Stegen et al., 2015). However, the short half-lives and complicated structures of these growth factors make them easy to degrade and deactivate. Alternatively, nickel hydroxide nanoparticles immobilized on the micro-/nanostructured surfaces of PEEK implants can facilitate the migration, tube formation, and angiogenic gene expression of human umbilical vein endothelial cells (HUVECs; Figure 2D; Dong et al., 2020). Apart from applying these osteogenic and angiogenic organic constituents, a recent study provided a novel direction for future research on genes such as human bone morphogenetic protein-4 (hBMP-4) to enhance bone regeneration (Cui et al., 2020).

## Modifications Inspired by The Topography of Bone

### Modifications of Surface Roughness

**Figure 3:**
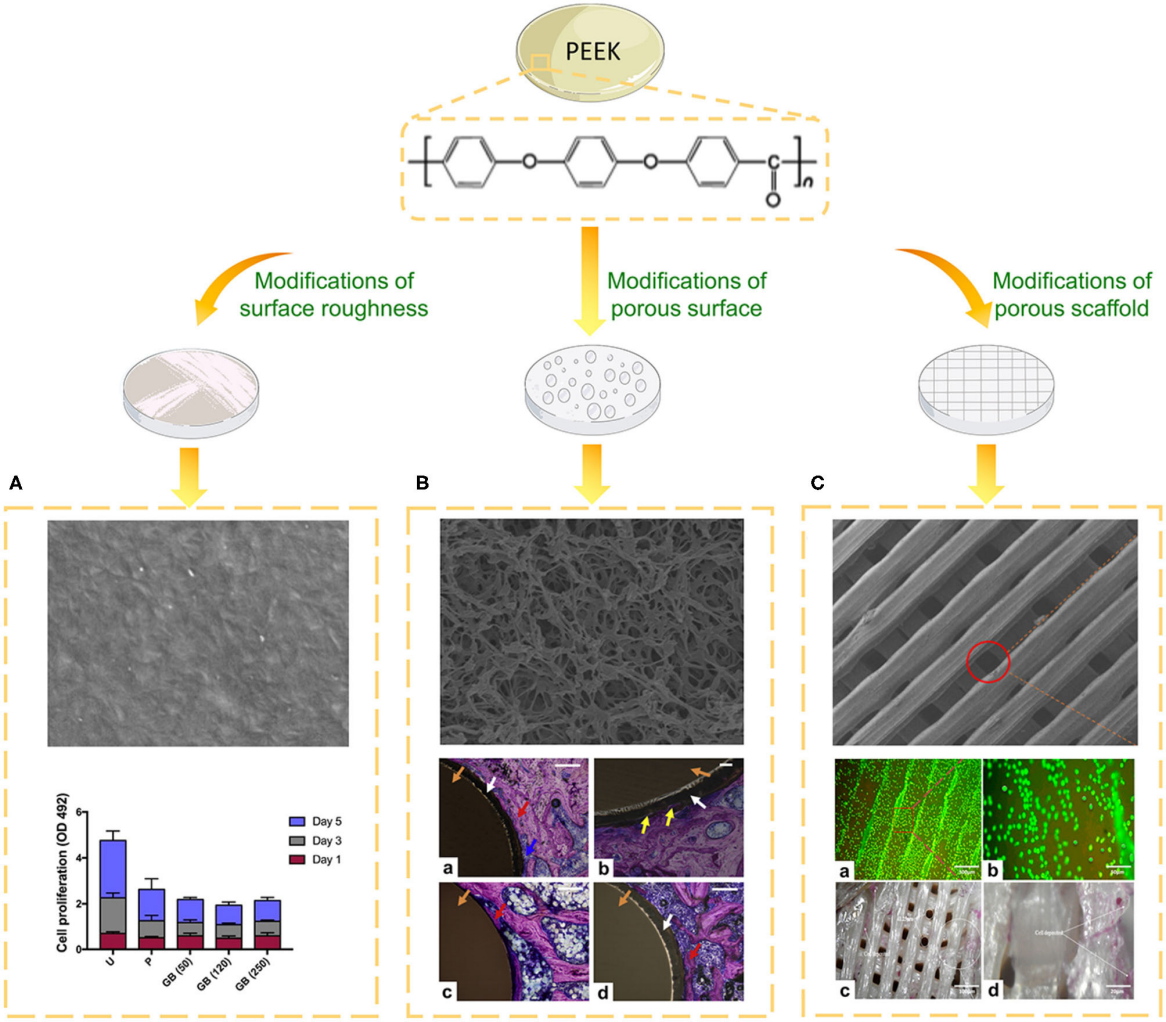
Biological evaluation of PEEK implants with different topographies. **(A)** SEM image of the surface of PEEK formed via fused filament fabrication and the cell proliferation of different groups on days one, three, and five. U, untreated; P, polished, GB (50), 50 μm grit-blasted; GB (120), 120 μm grit-blasted; and GB (250), 250 μm grit-blasted (Han et al., 2019a). **(B)** SEM image of the surface of sPEEK-WA and Giemsa-stained hard tissue around the implant at 8 weeks after implantation. Red arrows represent the newly formed bone, yellow arrows represent bone ingrowth, blue arrows represent osteoblasts, white arrows represent the sPEEK layer, and orange arrows represent the PEEK implant. (a) PEEK, (b) sPEEK-W, (c) sPEEK-A (low magnification), and (d) sPEEK-WA (high magnification). Scale bar is 200 μm. Reproduced from Zhao et al. (2013). Copyright (2013), with permission from Elsevier. **(C)** SEM image, cell spreading (a,b) and ALP activity (c,d) of the PEEK/cHAp scaffold after cultivation for 14 days: (a) magnification of 300 μm, (b) magnification of 50 μm, (c) magnification of 100 μm, and (d) magnification of 20 μm. Reproduced from Oladapo et al. (2020). Copyright (2020), with permission from Elsevier.

Bone is an inhomogeneous 3D structure with complicated topography. Surface roughness plays a pivotal role in topographical modification due to its effect on bone formation and implant fixation. Proper surface roughness can promote the absorption extra-cellular matrix (ECM) proteins (e.g., vitronectin, fibronectin and collagen I) and benefit the adhesion of osteoblasts (Pashkuleva et al., 2010). Subsequently, the ECM proteins provide a RGD sequence which performs as a receptor for osteoblast membrane proteins (mainly Integrins) and then initiate cell spreading, proliferation and differentiation (Mavropoulos et al., 2013). For instance, untreated PEEK with high surface roughness formed via fused filament fabrication displayed remarkably higher osteoblast proliferation and metabolic activity compared to grit-blasted PEEK with a smooth surface (Figure 3A; Han et al., 2019a). However, when the surface roughness exceeded 2.19 μm, osteoblast adhesion was inhibited because it is difficult to form osteoblastic pseudopodia between the larger crests and grooves (Anselme et al., 2000).

Microscale to nanoscale surface topography has been shown to affect osteoblastic cell adhesion. Micrometer-scale surface modifications (e.g., sandblasting) of PEEK implants have been used to enhance MG-63 cell behavior and bone-bonding ability (Deng et al., 2015a). Spraying PEEK surfaces with Ti plasma generated hierarchical roughness and increased the surface area (by 40% at the microscale), resulting in enhanced cell proliferation and accelerated cement line formation in hFOB 1.19 cells (Hickey et al., 2019). Compared to micro-roughness surfaces, nano-roughness surfaces showed greater initial protein (e.g., vitronectin and fibronectin) absorption, which subsequently mediates cell adhesion (Khang et al., 2007).

To date, several processes have been used to make nanoscale modifications to the surfaces of PEEK implants. The incorporation of nanosized particles like nHA via spin-coating can enhance the wettability, removal torque, and biocompatibility of PEEK (Johansson et al., 2014). The addition of nanoscale TiO_2_ (nTiO_2_) to PEEK via powder mixing and compression molding demonstrated that the enhanced surface roughness effectively promoted cell attachment and new bone regeneration (Wu et al., 2012). Other modifications including treatment with nitrogen, water, and ammonia PIII also displayed similar biological effects on PEEK (Lu et al., 2015; Zhao et al., 2016). Furthermore, the combination of oxygen plasma treatment and sandblasting imparted the PEEK surface with a micro/nano-topographical structure, which triggered the osteogenic differentiation of MG-63 cells and new bone formation (Xu et al., 2015).

### Modifications of the Porous Structure

Mimicking the morphology of trabecular bone is a crucial concept in the development of porous implant surfaces. Compared to rough surfaces, porous PEEK surfaces exhibit increased osteoblastic differentiation and bonding strength with bone (Torstrick et al., 2018, 2020). Numerous techniques have been applied to develop porous structures on the PEEK surface, including sulfonation, melt extrusion, porogen templating, and PIII technique (Zhao et al., 2013; Lu et al., 2014; Evans et al., 2015; Torstrick et al., 2016; Hieda et al., 2017; Yabutsuka et al., 2017; Deng L.-J. et al., 2018; Wu et al., 2018; Yuan et al., 2018; Conrad and Roeder, 2020; Swaminathan et al., 2020; Wan et al., 2020). Cell viability evaluations revealed that the optimal sulfonation treatment time was 5 min (Ma R. et al., 2020). Sulfonation can generate a 3D porous structure along with -SO_3_H groups, which are beneficial for pre-osteoblast functions, apatite formation, and bone growth (Figure 3B; Zhao et al., 2013). It's worth noting that sulfonation treatment can introduce more micropores and -SO_3_H groups on PEKK than PEEK for the reason that PEKK prossesses more ketone groups (Yuan et al., 2018). To further reinforce the bioactivity of porous PEEK, promising drug loading platforms were created by adding gelatin, hydrogel, chitosan, or polydopamine on the surface of PEEK (Deng L.-J. et al., 2018; Ouyang et al., 2018; Wu et al., 2018; Wang et al., 2019). For instance, poly(L-lactic acid)/simvastatin-loaded PEEK coated with a hyaluronic acid hydrogel enhanced the osteogenic differentiation of MC3T3-E1 and the expression of VEGF mRNA compared to uncoated PEEK (Deng L.-J. et al., 2018).

In recent years, clinical interest in 3D printing has grown rapidly. Techniques such as selective laser sintering (SLS) and fused filament fabrication have been adopted to construct PEEK scaffolds with controlled pore size (Roskies et al., 2016; Shuai et al., 2016; Deng et al., 2017; Peng et al., 2017; Oladapo et al., 2020; Spece et al., 2020). Up to now, there has been little consensus on the optimal pore size of scaffolds. Results revealed that porous PEEK with pore sizes ranged 200 to 508 μm exhibited higher proliferation and mineralization compared with smooth PEEK and Ti6Al4V (Torstrick et al., 2016). Another study presented that PEEK scaffold with 450 μm pore size displayed improved ingrowth of new bone and vascular perfusion (Feng et al., 2020). Although developed pore formation processes provide the ability to control the pore structures of PEEK implants, a deeper understanding of how pore size affects cell and tissue responses is needed. To further strengthen the biological properties of scaffolds for bone tissue engineering applications, HA and calcium HA were introduced into the PEEK matrix (Figure 3C; Vaezi et al., 2016; Oladapo et al., 2020). To reinforce the interfacial bonding between HA and PEEK, graphene oxide (GO) can be employed as an interfacial phase (Peng et al., 2017). Furthermore, the addition of graphene nanosheets, CNTs, or CF can boost the mechanical properties of PEEK/HA scaffolds (Shuai et al., 2016; Uddin et al., 2019; Swaminathan et al., 2020).

## Modifications Inspired by Bone Mechanical Function

Bone mainly consists of outer cortical bone and inner cancellous bone. The high modulus (16–23 GPa) of cortical bone endows it with stability and allows it to support the inner porous structure (Augat and Schorlemmer, 2006). The elastic modulus of PEEK (3–4 GPa) is close to that of human cortical bone, reducing the risk of high stress peaks and stress shielding effects during load transfer at the implant–bone interface. The stress shielding is a phenomenon which depicts stiff implants (e.g., titanium) cannot strain the surrounding bone adequately and then lead to bone resorption (Huiskes et al., 2000).

In consideration of the above, the mechanical properties of PEEK implants need to be elevated under specific clinical conditions. It's reported that CFs, CNTs, glass fibers (GFs), and graphene nanosheets (GNS) can be introduced into the PEEK matrix to satisfy the requirements and broaden its applications in load-bearing areas (Ji et al., 2015; Shuai et al., 2016; Yang et al., 2018; Han et al., 2019b). For instance, the stress distribution of a PEEK implant with 60% endless carbon fibers was similar to that of a titanium implant in dental application (Schwitalla et al., 2015). Mechanical property evaluations suggested that the elastic modulus of CF/PEEK and GF/PEEK could reach to 18 and 12 GPa, respectively (Lee et al., 2012). Furthermore, the mechanical properties of PEEK can be controlled by adding different amounts or lengths of fibers to the composite. For example, the bending strengths of CF/PEEK containing 25, 30, 35, and 40 wt% CF were 230.1–264.6 MPa, and the compressive strengths were 191.2–215.8 MPa (Qin et al., 2019). Meanwhile, the mechanical strengths of CF/PEEK composites containing CFs with lengths of 2–3 mm were more than two times those of the composites containing CFs with lengths of 150–200 μm (Li et al., 2019b). While fiber-reinforced PEEK has been manufactured through the traditional injection molding process, a novel technique called fused deposition modeling (FDM) has been applied currently. CF/PEEK formed via FDM was found to possess similar strength and toughness as the injection-molded samples along with better tensile and bending strengths compared to pure PEEK (Han et al., 2019b; Li et al., 2019a).

Although reinforced PEEK performs excellent mechanical properties, further investigations are needed to increase the bioactivity of hybrid PEEK materials. Researchers have proposed numerous hybrid materials containing different bioactive components, their mechanical and biological properties are summarized in Table 1. Moreover, the modification of CF-reinforced PEEK surfaces via chemical treatment, graphene functionalization, and PrA treatment has resulted in similar biological properties (Miyazaki et al., 2017; Yabutsuka et al., 2018; Yan J. H. et al., 2018; Ma J. et al., 2020).

**Table 1:** Mechanical and biological properties of carbon-based PEEK compounds.

**Composite**	**Content**	**Technique**	**Mechanical properties**	**Biological properties**	**References**
HA/CF/PEEK	Not reported	Plasma spray coating	Not reported	Enhanced the bone response to PEEK implants *in vivo*	Suska et al., 2014
HA/GNS+CNT/PEEK	10 wt% HA; 1 wt% GNS+CNT (weight ratio = 2:8)	SLS	Compressive strength = 78.65 MPa; compressive modulus = 4.79 GPa	Improved apatite-forming ability; enhanced cell adhesion, spreading, and proliferation	Shuai et al., 2016
nHA/CF/PEEK	25 wt% nHA; 20 wt% CF	Compounding and injection molding	Elastic modulus = 16.5 ± 0.7 GPa	Promoted MG63 cell attachment, proliferation, and osteogenic differentiation; enhanced calcium nodule formation and osseointegration	Deng et al., 2015b
nHA/multiwalled CNTs/PEEK	15 vol % nHA; 1.88 vol % CNTs	Melt-compounding and injection molding	Elastic modulus = 7.13 GPa; tensile strength = 64.48 MPa; elongation at break = 1.74%	Enhanced attachment, proliferation, differentiation, and mineralization of MC3T3-E1 cells	Liu et al., 2016
nTiO_2_/CF/PEEK	30 vol% CF	PIII	Improved elastic recovery and acceptable stability	Improved adhesion, proliferation, and osteoblastic differentiation of rBMSCs; imparted partial antibacterial activity against *S. aureus* and *E. coli*	Lu et al., 2014
GO/CF/PEEK	25 wt% and 40 wt% CF	Immersed in GO aqueous solution	Not reported	Enhanced adhesion, proliferation, ALP activity and mineralization of rBMSCs; promoted new bone formation *in vivo*	Qin et al., 2020
Amino groups/CF/PEEK	30 vol% CF	Plasma-enhanced chemical vapor deposition (PECVD)	Not reported	Promoted adhesion, proliferation, and osteogenic differentiation of MG-63 cells	Yu et al., 2020

## Modifications Inspired by Bone Immune Function

**Figure 4:**
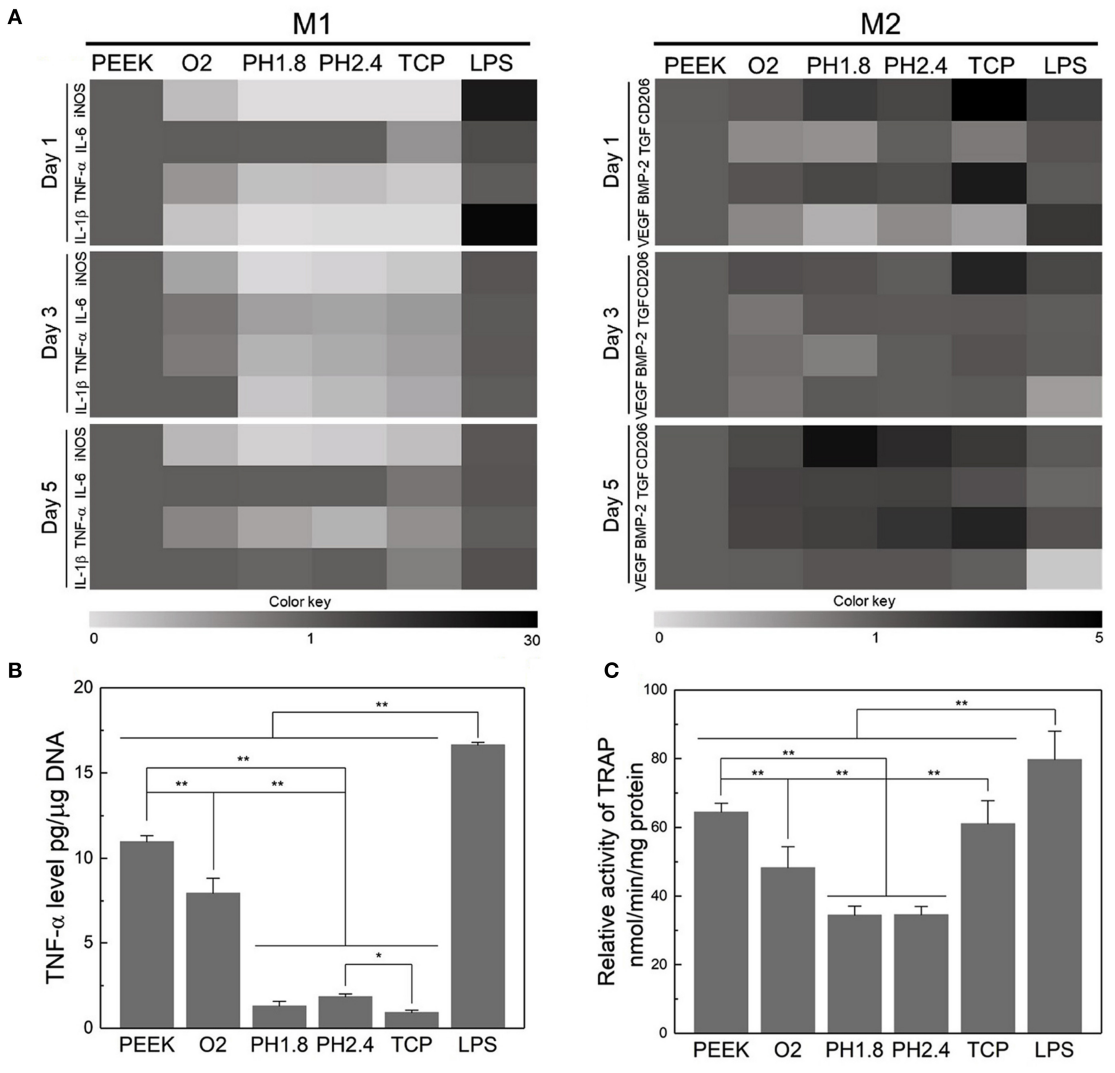
Evaluation of the acute inflammatory responses of RAW264.7 cells on different samples. **(A)** Heat map of the RT-PCR results of M1- and M2-related genes after incubation for 1, 3, and 5 days. **(B)** Level of TNF-α released by RAW264.7 cells after culturing for three days. **(C)** TRAP activity after culturing for 8 days to evaluate the osteoclastic differentiation of RAW264.7 cells. *p < 0.05, **p < 0.01. Reproduced from Gao et al. (2020). Copyright (2020), with permission from Elsevier.

Upon implantation, the innate immune response of bone tissue is mainly initiated by macrophages, which release a series of cytokines and growth factors. Macrophages can be divided into the pro-inflammatory M1 phenotype and anti-inflammatory M2 phenotype (Mosser and Edwards, 2008). To modulate the inflammatory response to PEEK implants, Gao et al. proposed a layer-by-layer self-assembly technique that facilitates the polarization of macrophages into the M2 phenotype and contributes to the upregulation of osteogenesis and the downregulation of osteoclastogenesis (Figure 4; Gao et al., 2020). Fukuda et al. found that the plasma treatment and subsequent phosphorylation on the PEEK surface attenuated the phenotypic polarization of RAW264.7 macrophages to an inflammatory phenotype (Fukuda et al., 2019). A recent study revealed that PEEK modified with hydrofluoric acid and nitric acid could promote the polarization of macrophages (M2 phenotype) and inhibit the expression of proinflammatory factors via the NF-κB pathway (Huo et al., 2020).

**Figure 5:**
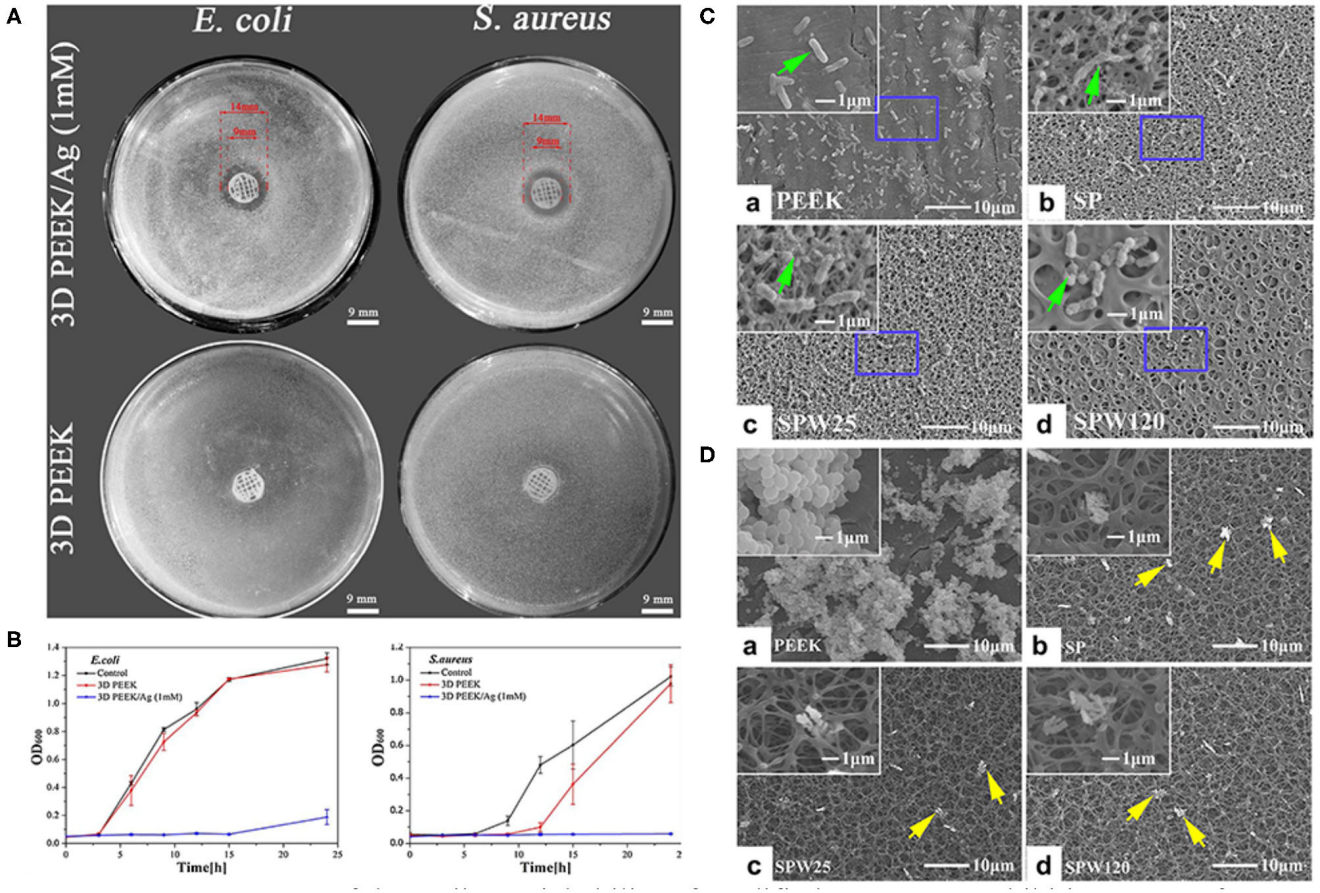
Assessment of the antibacterial ability of modified PEEK. **(A)** Inhibition zones of 3D PEEK and 3D PEEK/Ag against *E. coli* and *S. aureus*. **(B)** Bacterial dynamic curves of *E. coli* and *S. aureus* on 3D PEEK and 3D PEEK/Ag at OD600. Reproduced from Deng et al. (2017). Copyright (2017), with permission from Elsevier. **(C)** *E. coli* and **(D)** *S. aureus* seeded on the PEEK, SP (sulfonated only), SPW25 (sulfonated and hydrothermal treated at 25°C), and SPW120 (sulfonated and hydrothermal treated at 120°C) at a bacteria concentration of 10^7^ cfu/ml. The green arrows indicate the morphology of *E. coli* (high magnification), while the yellow arrows show the morphology of *S. aureus* (low magnification). Reproduced from Ouyang et al. (2016). Copyright (2016), with permission from Elsevier.

Researchers have also focused on surface modifications that target microbial infections. To the best of our knowledge, there are two ways to fabricate an anti-biofilm surface: depositing antibacterial coatings that release antimicrobial constituents or depositing antiadhesive coatings that restrict the adhesion of bacteria. Various antibiotics (e.g., lawsone, dexamethasone, minocycline, hinokitiol, tobramycin, gentamicin sulfate, and antimicrobial peptides) have been adopted to treat infections caused by implants (Ur Rehman et al., 2018; Zhang et al., 2018b; Deng et al., 2019; Xu et al., 2019; Yuan et al., 2019; Xue et al., 2020; Yin et al., 2020). Since the abuse of antibiotics has contributed to the emergence of bacterial resistance, several ions and their nanoparticles have emerged as promising alternatives to antibiotics (Figures 5A,B). The details of the techniques involved and the biological effects of these ions are summarized in Table 2. It is worth noting that the uncontrolled release of bactericides will exacerbate bacterial resistance. To avoid this phenomenon, silk fibroin, which was reported to cause the pH-responsive controlled release of bactericides, has been introduced into PEEK implants (Yan J. et al., 2018).

**Table 2:** Summary of studies on antibacterial ions and their biological effects.

**Ions**	**Type**	**Technique**	**Biological effects**	**References**
Sulfur	Concentrated sulfuric acid (95–98%)	Sulfonation followed by hydrothermal treatment	Enhanced proliferation and osteogenic differentiation of rBMSCs; improved osseointegration; generated antibacterial properties against *E. coli* and *S. aureus*	Ouyang et al., 2016
Fluorine	Hydrofluoric acid	PIII followed by hydrofluoric acid treatment	Enhanced rBMSCs adhesion, spreading, proliferation, ALP activity, and osseointegration; resulted in a good bacteriostatic effect against *Porphyromonas gingivalis*	Chen et al., 2017
Nitrogen	N_2_	PIII	Enhanced biological activity and antibacterial activity against *S. aureus*	Gan et al., 2016
Selenium	Red selenium nanoparticles and gray selenium nanorods	Quick precipitation	Inhibited the growth of *Pseudomonas aeruginosa*	Wang Q. et al., 2016
Silver	Silver nitrite	Soft-solution process	Generated strong antibacterial activity against *S. aureus* and *E. coli*	Kakinuma et al., 2015
	Nanoparticles	Catecholamine chemistry	Resulted in a significant antibacterial effect against *S. aureus* and *E. coli*; enhanced MG63 cell proliferation and ALP activity	Deng et al., 2017
	Nano-silver	Magnetron sputtering technology	Significantly increased surface roughness and water contact angle; improved antibacterial activity against *S. mutans* and *S. aureus*; improved the bacterial adhesion ability of PEEK	Liu et al., 2017
	Nanoclusters	Radio frequency co-sputtering	Generated antibacterial effects against *E. coli* and *S. carnosus*	Ur Rehman et al., 2017
Copper	Nanoparticles	Magnetron sputtering	Produced a bactericidal effect against methicillin-resistant *S. aureus* (MRSA); activated macrophage polarization to a pro-inflammatory phenotype with improved phagocytosis for MRSA	Liu et al., 2019
ZnO	Nanopowder	Melt-blending	Improved the mechanical and tribological properties; resulted in superior antibacterial activity against *S. aureus* and *E. coli*	Díez-Pascual and Díez-Vicente, 2014
Ag/ZnO	Nanoparticles	Sulfonation and layer-by-layer self-assembly	Inhibit the reproduction of *E. coli* and *S. aureus*; enhanced the adhesion, proliferation, and spreading of MG-63 cells; increased ALP activity and the levels of osteogenesis-related genes	Deng Y. et al., 2018
Titanium	Titanium ions	PIII	Promoted the adhesion, proliferation, and osteoblastic differentiation of bMSCs; generated partial resistance to *S. aureus* and *E. coli*	Lu et al., 2014
	TiO_2_ nanoparticles	PIII	improved the adhesion, migration, proliferation, and collagen secretion ability of HGFs; produced antibacterial properties against *Streptococcus mutans, Fusobacterium nucleatum*, and *P. gingivalis*	Wang X. et al., 2016
Magnesium	Particles	Vapor deposition method	Strongly killed *S. aureus* with anti- bacterial rate reaching to 99%	Yu et al., 2018
Si_3_N_4_	Powder	Melt blending	Significantly improved osteoconduction in SAOS-2 cells; improved the bacteriostatic properties against *Staphylococcus epidermidis*	Pezzotti et al., 2018
Zinc-magnesium silicate	Nanopowder	Melt blending	Improved the mechanical properties, surface roughness, hydrophilicity, and apatite mineralization ability; enhanced the attachment, proliferation, and differentiation of MC3T3-E1 cells; inhibited the growth of *E. coli*	Tang et al., 2019

Surface roughness and hydrophobicity are the dominant factors affecting bacterial adhesion and biofilm formation. Bollen et al. suggested that enhancing the surface roughness can improve the attachment of bacteria when Ra >0.2 μm (Bollen et al., 1996). In addition, it has been reported that bacteria tend to adhere on implants with hydrophilic surfaces (Das et al., 2010). To prepare porous surfaces, sulfonation followed by hydrothermal treatment which can remove residues are applied (Ouyang et al., 2016; Yuan et al., 2019). Moreover, a porous surface can limit the adhesion of bacteria with different shapes and sizes. For instance, porous structures are considered to be more easily to trap spherical *S. aureus* compared to elongated *E. coli* (Figures 5C,D; Ouyang et al., 2016).

Except for microbial infections, bone tumor like osteosarcoma should also be taken into consideration. In order to reduce the risk of tumor recurrence after a bone removal surgery, applying an implant with anti-cancer agents is an effective method. Studies confirmed that the anti-cancer drugs (e.g., methotrexate, and 5-fluorouracil) loaded in ceramic cements could inhibit the growth of osteosarcoma cell line (Prasad et al., 2018; Wu et al., 2020). Currently, light-assisted photothermal therapy (PTT) based on photothermal conversion agent (e.g., MXenes) was introduced to PEEK implants to defect osteosarcoma cells (Yin et al., 2020).

## Conclusion

After decades of research, PEEK implants in bone tissue engineering have experienced three stages of development: (1) first-generation implants possessing outstanding mechanical properties; (2) second-generation implants with prominent cytocompatibility and osteogenic activity; and (3) third-generation implants equipped with outstanding osseointegration and anti-inflammation/-infection ability. According to previous studies, we have grouped PEEK modifications into four categories based on their inspiration: the constituents (inorganic phase, mineral ions, and organic phase), structure (surface roughness and porosity), mechanical function, and immune function of human bone.

Although the techniques for modifying PEEK have matured, numerous remaining obstacles must be overcome before modified PEEK implants can be applied in clinical practice: (1) the ratio of different constituents must be optimized to maximize bone regeneration and the mechanical properties; (2) methods must be developed to control the release of biomolecules and achieve the desired long-term effects; and (3) experiments are needed to identify the ideal pore structure to optimize biological performance. Thus, extensive studies are still needed to observe the long-term implantation of PEEK *in vivo* and elucidate which strategies are appropriate to modify PEEK in clinical settings. In this respect, the complexity, reproducibility, stability and cost-effectiveness of the manufacturing process should also be considered. In the future, researchers will focus on PEEK implants possessing the optimal combination of osteointegration, vascularization, anti-inflammation/-infection, and mechanical properties. It is worth noting that modifications that mimic the components, structure, and function of human bone are expected to be the key to fabricating multifunctional implants. Also, it can provide inspirations for researchers to modify other PAEK materials and expand their applications in the field of orthopedic, spinal and dental impalnts.

## Author Contributions

XG conceptualized and designed the manuscript. XG and XS drafted the manuscript. YS and JW designed the table. YL, KY, and YW designed the figure. YZ revised the manuscript. All authors approved the final version.

## Conflict of Interest

The authors declare that the research was conducted in the absence of any commercial or financial relationships that could be construed as a potential conflict of interest. The reviewer YZ declared a shared affiliation, with no collaboration, with the authors to the handling Editor at the time of the review.
